# Novel Environmentally-Friendly Process for Selective Extraction and Enrichment of DHA/EPA-Containing Phospholipids from Krill Oil via Differential Temperature-Controlled Crystallization

**DOI:** 10.3390/foods14162841

**Published:** 2025-08-16

**Authors:** Yi He, Yu Zhang, Jiangying Heng, Bo Liu, Xuan Ma, Jing Jin, Wenjie Yan, Feng Wang

**Affiliations:** 1 College of Biochemical Engineering, Beijing Union University, Beijing 100101, China; hy1303356491@163.com (Y.H.);; 2 Beijing Key Laboratory of Bioactive Substances and Functional Food, Beijing Union University, Beijing 100101, China

**Keywords:** DHA/EPA-containing phospholipids, green extraction method, differential crystallization, response surface methodology, molecular species distribution, oxidative stability

## Abstract

This study presents a novel environmentally-friendly process for the selective extraction and enrichment of DHA/EPA-containing phospholipids (PL-DHA/EPA) from krill oil. The methodology leverages differential crystallization behavior between phospholipids and triacylglycerols in ethanolic solutions, exploiting their distinct freezing point thresholds to achieve precise fractionation. Response surface methodology optimization identified optimal extraction parameters: liquid-to-material ratio of 6:1 (*v*/*w*), freezing temperature of −20 °C, freezing duration of 25 h, and rotary evaporation temperature of 45 °C, yielding a final product with 39.40% PL-DHA/EPA content. Principal component analysis revealed substantial overlap in confidence ellipses among extraction methodologies, indicating effective preservation of core phospholipid signatures from the parent krill oil while maintaining critical structural characteristics and molecular species distribution. Comprehensive analysis of phospholipid fractions and heatmap analysis revealed distinctive molecular profiles compared to conventional organic solvent extraction, with selective enrichment of EPA-containing phospholipids, particularly PC-EPA and PI-EPA species. The green extraction method demonstrated comparable oxidative stability to conventional approaches, with superior protection against secondary oxidation as evidenced by significantly lower anisidine values. This sustainable approach achieves effective phospholipid enrichment while substantially reducing environmental impact through elimination of halogenated solvents, addressing the critical need for environmentally conscious technologies in marine lipid processing with potential applications in nutraceutical and functional food industries.

## 1. Introduction

Antarctic krill (*Euphausia superba*) represents a pivotal marine crustacean species inhabiting the Southern Ocean, constituting one of Earth’s most abundant biomass resources with an estimated standing stock exceeding 500 million tons. These small planktonic organisms serve as fundamental components of marine food webs while offering exceptional nutritional value through their unique lipid composition characterized by high concentrations of omega-3 fatty acids in phospholipid forms [1]. Krill oil (KO) has emerged as a superior source of marine-derived bioactive compounds, particularly distinguished by its unique phospholipid-containing omega-3 fatty acids that demonstrate enhanced bioavailability compared to conventional fish oil sources.

The predominant bioactive components in KO include docosahexaenoic acid (DHA) and eicosapentaenoic acid (EPA), which exist primarily in phospholipid forms, marking a significant difference from conventional fish oils, where these fatty acids are mainly present as triglycerides [2]. DHA serves critical roles in neural development, cognitive function, and retinal health through its incorporation into neuronal membrane phospholipids, where it facilitates optimal membrane fluidity and supports synaptic transmission [3]. EPA functions as a precursor to specialized pro-resolving mediators, including resolvins and protectins, which actively resolve inflammatory processes and promote tissue homeostasis [4]. The phospholipid-bound forms of these fatty acids demonstrate superior bioavailability, with absorption rates 2–3 times higher than triglyceride forms, attributed to their enhanced solubility and direct incorporation into cellular membranes.

DHA/EPA-Containing Phospholipids (PL-DHA/EPA) demonstrate enhanced bioavailability and superior cellular incorporation compared to their triglyceride counterpart. This structural advantage stems from the amphipathic nature of phospholipids, facilitating improved absorption across biological membranes through specific transport mechanisms, including the lysophosphatidylcholine transporter MFSD2A, which enables direct blood-brain barrier penetration [5]. Furthermore, PL-DHA/EPA exhibits remarkable stability against oxidation due to the protective microenvironment created by polar headgroups and demonstrates unique biological activities, including enhanced brain delivery and superior anti-inflammatory properties through preferential incorporation into membrane rafts and signaling complexes [6].

Traditional extraction methods for isolating PL-DHA/EPA from krill oil primarily rely on organic solvent-based processes, which present environmental concerns and potential safety risks. Conventional techniques, such as Folch extraction and the Bligh-Dyer method, while effective, utilize substantial quantities of chlorinated solvents and require multiple separation steps. Recent advances in green chemistry have highlighted the necessity for developing sustainable extraction protocols that minimize environmental impact while maintaining extraction efficiency [7]. Current research trends focus on developing environmentally-friendly extraction technologies that optimize PL-DHA/EPA recovery while adhering to green chemistry principles. Emerging approaches include supercritical fluid extraction, enzyme-assisted extraction, and ionic liquid-based methods. However, these techniques often face challenges in the selective enrichment of DHA/EPA-containing phospholipids and scale-up feasibility.

This study presents a novel environmentally-friendly process for the selective extraction and enrichment of PL-DHA/EPA from krill oil through differential temperature-controlled crystallization in ethanolic systems. The proposed methodology leverages the differential crystallization behavior between phospholipids and triacylglycerols in ethanolic solutions, exploiting their distinct freezing point thresholds to achieve precise fractionation. This temperature-dependent fractionation enables selective precipitation of triacylglycerols at −20 °C while maintaining phospholipid solubilization, achieving high-purity PL-DHA/EPA recovery through sustainable solvent systems. The research addresses the critical need for efficient, scalable, and environmentally conscious extraction technologies in marine lipid processing.

## 2. Materials and Methods

### 2.1. Materials

Krill oil was purchased from Feist Biotechnology Co., Ltd. (Xi’an, China). Anhydrous ethanol (analytical grade, ≥99.5%) was purchased from Sigma-Aldrich (St. Louis, MO, USA). All other chemicals and solvents used were of analytical grade.

### 2.2. Separation and Purification of PL-DHA

#### 2.2.1. Extraction Procedure

Krill oil samples were precisely weighed and mixed with anhydrous ethanol according to the designed ratios. The mixtures were subjected to magnetic stirring at 500 rpm under controlled temperature conditions specified by the experimental design. Following extraction, the suspensions were centrifuged at 5000× *g* for 15 min at 4 °C. The supernatants containing PL-DHA/EPA were collected for subsequent purification.

#### 2.2.2. Purification Process

The obtained supernatants underwent low-temperature crystallization at −20 °C for 12 h to separate triglycerides from phospholipids. The crystallized triglycerides were removed by centrifugation (8000× *g*, 20 min, −10 °C). The phospholipid-enriched fractions were concentrated using rotary evaporation (40 °C, −0.1 MPa) to yield purified PL-DHA/EPA.

#### 2.2.3. Experimental Design

The optimization of the PL-DHA/EPA extraction process was conducted through a systematic investigation of process parameters. Initially, single-factor experiments were performed to evaluate the effects of critical processing variables on both phospholipid content and PL-DHA/EPA yield in the purified product. The investigated parameters included liquid-to-material ratio (ranging from 4:1 to 8:1 with an interval of 1:1), freezing temperature (from −30 °C to 10 °C with 10 °C increments), freezing duration (from 10 h to 30 h with 5 h increments), and rotary evaporation temperature (from 25 °C to 65 °C with 10 °C increments).

Building upon single-factor experimental results, a response surface methodology (RSM) was implemented to optimize the extraction process and explore the interactive effects among process variables. A Box-Behnken experimental design was constructed using Design Expert 13 software, with PL-DHA/EPA yield (Y) serving as the response variable. The independent variables consisted of the aforementioned four parameters: liquid-to-material ratio (A), freezing temperature (B), freezing duration (C), and rotary evaporation temperature (D). The coded and actual levels of these variables are detailed in Table 1.

**Table 1:** Design of factors and levels of response surface test.

A/(*v*/*v*)	B/(°C)	C/(h)	D/(°C)
5	−30	20	35
6	−20	25	45
7	−10	30	55

#### 2.2.4. Response Surface Statistical Analysis

The experimental data were fitted to a second-order polynomial model:

Y = β_0_ + ΣβᵢXᵢ + ΣβᵢᵢXᵢ^2^ + ΣβᵢⱼXᵢXⱼ

where Y represents the response variable (PL-DHA yield), β_0_ is the intercept coefficient, βᵢ, βᵢᵢ, and βᵢⱼ are the linear, quadratic, and interaction coefficients, respectively. Analysis of variance (ANOVA) was performed to evaluate the statistical significance of the model terms (*p* < 0.05).

#### 2.2.5. Model Validation

The optimized conditions predicted by the RSM model were validated through triplicate experiments. The experimental values were compared with the predicted responses to verify the accuracy and reliability of the established model.

### 2.3. Organic Solvent Extraction of PL-DHA/EPA

PL-DHA/EPA extraction was performed using a modified Folch method employing a chloroform-methanol biphasic system optimized [8]. Accurately weighed krill oil samples (5.0000 ± 0.0001 g) were homogenized with 100 mL of chloroform-methanol solution (2:1, *v*/*v*) using a high-speed homogenizer at 10,000 rpm for 3 min under a nitrogen atmosphere to prevent oxidative degradation during extraction. The homogenized mixture was transferred to a separatory funnel and allowed to stand for 30 min at room temperature to achieve phase equilibrium. Subsequently, 20 mL of distilled water was added to induce phase separation, followed by gentle mixing and standing for an additional 15 min until clear phase demarcation was observed. The lower chloroform phase containing dissolved phospholipids was carefully collected through the bottom outlet, while the upper methanol-water phase containing polar impurities was discarded. The chloroform extract was subjected to anhydrous sodium sulfate treatment to remove residual moisture, followed by filtration through Whatman No. 1 filter paper to eliminate particulate matter. Solvent removal was accomplished using rotary evaporation at 40 °C under reduced pressure (20 mbar) with a nitrogen blanket to prevent thermal degradation and oxidative stress on polyunsaturated fatty acid chains. The resulting phospholipid concentrate was redissolved in minimal chloroform volume and transferred to pre-weighed amber vials for nitrogen flushing and storage at −20 °C until analysis.

### 2.4. Lipidomic Analysis Determination

Comprehensive lipidomic analysis was performed using ultra-high-performance liquid chromatography coupled with high-resolution mass spectrometry (UHPLC-QExactive Plus Orbitap, ThermoFisher Scientific, Waltham, MA, USA) to quantify PL-DHA and EPA species. Sample preparation involved dissolution of extracted phospholipid concentrates in chloroform-methanol (1:1, *v*/*v*) to achieve final concentrations of 1 mg/mL, followed by filtration through 0.22 μm PTFE syringe filters to remove particulate matter prior to instrumental analysis.

Chromatographic separation was achieved using a ZORBAX Eclipse Plus C18 RRHD column (1.8 μm, 3.0 × 150 mm, Agilent Technologies, Santa Clara, CA, USA) maintained at 40 °C to ensure optimal peak resolution and reproducible retention times. The gradient elution employed mobile phase A (acetonitrile-water, 6:4, *v*/*v*, containing 10 mM formic acid) and mobile phase B (acetonitrile-isopropanol, 1:1, *v*/*v*, containing 10 mM formic acid). The gradient program initiated at 30% mobile phase B, increased linearly to 100% B over 15 min, maintained at 100% B for 5 min, then returned to initial conditions for column re-equilibration over 3 min. Flow rate was maintained at 0.3 mL/min with an injection volume of 2 μL to achieve optimal sensitivity while preventing column overloading.

Mass spectrometric detection was performed using a Q Exactive Plus Orbitrap mass analyzer operating in data-dependent acquisition mode with polarity switching between positive and negative electrospray ionization. The instrument parameters included spray voltage of 3.5 kV, capillary temperature of 350 °C, sheath gas flow rate of 35 arbitrary units, and auxiliary gas flow rate of 10 arbitrary units. Full MS scans were acquired at 70,000 resolution over m/z range 100–1000 with automatic gain control target of 3 × 10^6^ and maximum injection time of 100 ms. Data-dependent MS^2^ fragmentation was triggered for the top five most abundant ions using normalized collision energy of 25, 30, and 35% stepped collision energy to generate comprehensive diagnostic fragment ions for structural confirmation.

The dd-MS^2^ acquisition utilized 17,500 resolution with AGC target of 1 × 10^5^, maximum injection time of 50 ms, and isolation window of 4.0 m/z to ensure adequate spectral quality for molecular identification. Dynamic exclusion was set to 10.0 s to prevent redundant fragmentation of the same precursor ions. Phospholipid molecular species identification was accomplished through accurate mass measurements with mass tolerance below 3 ppm, combined with characteristic fragmentation patterns including phospholipid headgroup-specific neutral losses and fatty acid chain identification through diagnostic fragment ions.

### 2.5. Peroxide Value Determination

Accurately weighed samples (0.5000 ± 0.0001 g) were dissolved in 10 mL of acetic acid-chloroform solution (3:2, *v*/*v*) under a nitrogen atmosphere, followed by the addition of 0.5 mL saturated potassium iodide solution with vigorous shaking for 30 s. The reaction mixture was incubated in darkness for 5 min at room temperature to ensure quantitative iodine liberation from lipid hydroperoxides. Subsequently, 25 mL of distilled water was added to halt the reaction and facilitate phase separation for titration analysis. The liberated iodine was quantified through titration with standardized 0.01 N sodium thiosulfate solution using starch indicator (1% *w*/*v*) until complete disappearance of the blue-black complex. Blank determinations were performed simultaneously to account for background iodine content in reagents. Peroxide values were calculated using the established formula:

$$PV(gI_{2}/100g)=\left(S-B\right)*N*126.9/W$$

where *S* represents the sample titration volume, *B* denotes the blank titration volume, *N* indicates sodium thiosulfate normality, and *W* represents sample weight in grams. All determinations were performed in triplicate under controlled laboratory conditions to ensure analytical precision and reproducibility. The method detection limit was established at 0.05 g I_2_/100 g with relative standard deviation maintained below 3% for quality assurance purposes.

### 2.6. Acid Value Determination

Accurately weighed samples (1.0000 ± 0.0001 g) were dissolved in 25 mL of neutralized ethanol-diethyl ether solution (1:1, *v*/*v*) with gentle heating at 40 °C to ensure complete phospholipid dissolution. The sample solution was titrated with standardized 0.1 N potassium hydroxide ethanolic solution using phenolphthalein indicator (0.5 mL of 1% ethanolic solution) until a persistent pink coloration appeared for 30 s, indicating complete neutralization of free carboxylic acid groups. Blank determinations were conducted simultaneously using identical solvent volumes without sample addition to account for residual reagent acidity and atmospheric carbon dioxide absorption effects. Acid values were calculated using the established formula:

$$AV(mg\;KOH/g)=\left(S-B\right)*N*56.1/W$$

where *S* represents the sample titration volume, *B* denotes the blank titration volume, *N* indicates potassium hydroxide normality, and *W* represents sample weight in grams. All determinations were performed in triplicate under controlled laboratory conditions with relative standard deviation maintained below 2%.

### 2.7. Anisidine Value Determination

Accurately weighed samples (0.2500 ± 0.0001 g) were dissolved in 25 mL of isooctane, and 5 mL aliquots were mixed with 1 mL of *p*-anisidine reagent (0.25% *w*/*v* in glacial acetic acid). The reaction mixture was incubated at room temperature for exactly 10 min in darkness to allow quantitative formation of the colored Schiff base complex between aldehydic carbonyl groups and p-anisidine. Absorbance measurements were performed at 350 nm using a UV-visible spectrophotometer with sample blanks prepared using glacial acetic acid without p-anisidine to correct for background absorption. Anisidine values were calculated using the formula:

AV=25∗(1.2∗As−Ab)/m

where *As* represents sample absorbance with anisidine reagent, *Ab* denotes sample blank absorbance, and *m* indicates sample weight in grams. The factor 1.2 accounts for the dilution effect of anisidine reagent addition. All determinations were performed in triplicate under controlled lighting conditions with relative standard deviation maintained below 3%.

### 2.8. Total Oxidation (TOTOX) Value Determination

The total oxidation (TOTOX) value was calculated as a comprehensive oxidative deterioration index using the standard formula:

TOTOX=2∗PV+AV

where *PV* represents the peroxide value (g I_2_/100 g) and *AV* denotes the anisidine value. Both peroxide and anisidine values were determined simultaneously on identical sample batches to ensure temporal consistency.

### 2.9. Statistical Analysis

All experiments were conducted at least three times, with results expressed as mean ± standard deviation. Statistical analyses were performed using SPSS 17.0 (SPSS Inc., Chicago, IL, USA), employing one-way analysis of variance (ANOVA) followed by Duncan’s multiple range test as the post-hoc procedure. Statistical significance was established at *p* < 0.05.

## 3. Results and Discussion

### 3.1. Single-Factor Analysis of Process Parameters

#### 3.1.1. Effect of Liquid-to-Material Ratio on PL-DHA/EPA Extraction

**Figure 1:**
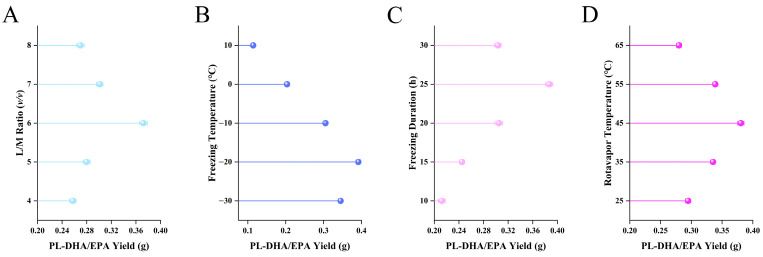
Single-factor analysis of critical parameters affecting PL-DHA/EPA extraction from Antarctic krill oil. (**A**) Effect of liquid-to-material ratio (*v*/*w*) on PL-DHA/EPA yield. (**B**) Effect of freezing temperature on PL-DHA/EPA yield. (**C**) Effect of freezing duration on PL-DHA/EPA yield. (**D**) Effect of rotary evaporation temperature on PL-DHA/EPA yield. PL-DHA/EPA yield represents the weight (g) of PL-DHA/EPA obtained from 10 g of krill oil. Error bars represent standard deviation (*n* = 3).

The influence of liquid-to-material ratio on PL-DHA/EPA yield was systematically investigated while maintaining constant extraction parameters (freezing temperature at −20 °C, freezing duration at 20 h, and rotary evaporation temperature at 45 °C) (Figure 1A). Experimental results demonstrated that PL-DHA/EPA yield exhibited a parabolic relationship with increasing liquid-to-material ratio, reaching optimal extraction efficiency at a ratio of 6:1.

Within the lower liquid-to-material ratio range (4:1 to 6:1), PL-DHA/EPA yield increased significantly with increasing ratios, attributable to enhanced mass transfer kinetics and improved phospholipid solubility [9,10]. The adequate solvent volume established a steeper concentration gradient, facilitating the migration of PL-DHA/EPA from the krill oil matrix into the solvent phase. Concurrently, appropriate solvent volume ensured optimal molecular collision frequency between solvent molecules and phospholipid components [11], thereby enhancing extraction efficiency from a molecular dynamics perspective. The polarity of the solvent, compatible with the amphiphilic characteristics of phospholipid molecules, further promoted selective dissolution and separation of target components.

Conversely, when the liquid-to-material ratio exceeded the threshold of 6:1, PL-DHA/EPA yield exhibited a declining trend. This phenomenon can be elucidated through multiple mechanistic pathways: excessive solvent volumes facilitated co-extraction of non-target substances (such as neutral lipids, pigments, and other impurities) [12], which subsequently interfered with PL-DHA/EPA isolation during the freezing crystallization and filtration stages. Moreover, elevated liquid-to-material ratios diluted the phospholipid concentration in the extraction system, reducing supersaturation during cold crystallization processes [13], thereby compromising the aggregation and precipitation kinetics of phospholipid molecules [14]. Notably, excessive solvent quantities may also induce conformational changes in phospholipid molecular structure, affecting their crystallization behavior under low-temperature conditions [15,16]. From a process engineering perspective, surplus solvent volumes increased energy consumption and operational complexity during subsequent concentration steps.

#### 3.1.2. Effect of Freezing Temperature on PL-DHA/EPA Extraction

The influence of freezing temperature on PL-DHA/EPA yield was examined while maintaining constant conditions (liquid-to-material ratio at 6:1, freezing duration at 20 h, and rotary evaporation temperature at 45 °C) (Figure 1B). As shown in Figure 1, PL-DHA/EPA yield exhibited a temperature-dependent pattern, with the highest yield achieved at −20 °C. At higher temperatures (10 °C to 0 °C), the insufficient thermal energy reduction resulted in incomplete separation of neutral lipids from phospholipids [17,18]. This can be attributed to the similar solubility characteristics of these lipid components at elevated temperatures, leading to poor selective crystallization [19]. When the temperature decreased to −10 °C, the yield increased markedly due to the enhanced crystallization of neutral lipids, particularly triglycerides, while phospholipids remained predominantly in solution.

The optimal yield observed at −20 °C can be mechanistically explained by two factors. Firstly, this temperature promotes efficient crystallization of neutral lipids without significantly affecting phospholipid solubility, owing to the distinct phase transition temperatures between these lipid classes. Secondly, at −20 °C, the formation of ordered crystal structures of neutral lipids facilitates their removal through filtration, resulting in enhanced PL-DHA/EPA recovery in the filtrate. However, further temperature reduction to −30 °C led to a decreased yield. This decline can be attributed to the partial crystallization of phospholipids, particularly those with saturated fatty acid moieties, which may co-precipitate with neutral lipids. Additionally, extremely low temperatures can alter the molecular arrangement of phospholipids in solution, potentially leading to their aggregation and loss during the filtration process (Pichot et al., 2013 [11]).

#### 3.1.3. Effect of Freezing Duration on PL-DHA/EPA Extraction

The temporal influence of freezing duration on PL-DHA/EPA extraction efficiency was methodically examined under standardized experimental parameters (liquid-to-material ratio: 6:1; freezing temperature: −20 °C; rotary evaporation temperature: 45 °C) (Figure 1C). Chromatographic quantification revealed a distinct time-dependent extraction profile, with PL-DHA/EPA yield reaching its apex at 25 h of cryogenic treatment.

During the initial crystallization regime (10 h to 20 h), PL-DHA/EPA yield exhibited a progressive augmentation, corresponding to the incremental solidification of neutral lipid constituents. This temporal window encompasses primary nucleation initiation and subsequent crystal propagation phenomena [20], wherein triglycerides and analogous neutral lipids undergo thermodynamically-driven phase transitions to form embryonic crystalline domains [21]. The crystallization kinetics within this interval, however, proved insufficient for comprehensive phase demarcation, resulting in diminished extraction efficiency due to incomplete solidification of contaminating lipid species. The molecular diffusion coefficients at this stage remain sufficiently elevated to permit dynamic rearrangement of lipid assemblies without achieving solid-liquid equilibrium, thereby impeding definitive separation between phospholipid and neutral lipid fractions [22].

The extraction efficiency culminated at 25 h, signifying the establishment of optimal crystallization equilibrium within the system. At this critical temporal threshold, the physicochemical environment facilitates comprehensive crystallization of neutral lipids, enabling their effective segregation via subsequent filtration methodologies. This duration permits adequate crystal maturation characterized by Ostwald ripening and secondary nucleation phenomena, yielding macroscopic crystalline architectures with enhanced thermodynamic stability and reduced interfacial energy [23]. Concomitantly, this timeframe facilitates optimal diffusional migration of phospholipid molecules away from the solidifying triglyceride matrix while preserving their supramolecular integrity. The rheological properties of the semi-crystalline suspension at this duration promote enhanced percolation of the phospholipid-enriched liquid phase through the interstices of the crystalline network, maximizing phospholipid recovery in the filtrate.

Extended cryogenic exposure (30 h) precipitated a discernible decline in extraction yield through multifaceted mechanisms. Prolonged low-temperature treatment induces gradual crystallization of phospholipid species possessing elevated phase transition temperatures, particularly those incorporating saturated acyl chains [24,25], resulting in their unintended co-precipitation with neutral lipids. Furthermore, extended crystallization durations promote polymorphic transitions within the crystal lattice from metastable α-forms to more stable β′ or β configurations with augmented molecular packing densities [26,27]. These structural reorganizations potentially occlude phospholipid molecules within the evolving crystal framework through hydrophobic interactions or physical entrapment mechanisms, consequently reducing their extractability. Additionally, prolonged freezing may disrupt phospholipid-solvent interactions, altering solvation shells and promoting phospholipid self-association into larger aggregates with reduced solubility [28]. The formation of mixed crystals (solid solutions) between neutral lipids and phospholipids also becomes thermodynamically favorable during extended crystallization periods, further compromising separation efficiency.

#### 3.1.4. Effect of Rotary Evaporation Temperature on PL-DHA/EPA Extraction

The thermodynamic influence of rotary evaporation temperature on PL-DHA/EPA recovery was systematically investigated while maintaining constant experimental parameters (liquid-to-material ratio: 6:1; freezing temperature: −20 °C; freezing duration: 25 h) (Figure 1D). Quantitative analysis revealed a distinct temperature-dependent extraction profile with maximal PL-DHA/EPA yield at 45 °C, demonstrating the critical role of thermal conditions in both solvent removal efficiency and molecular integrity preservation.

At suboptimal temperatures (25 °C to 35 °C), diminished recovery rates were observed, attributable to insufficient thermodynamic driving force for effective solvent volatilization. The reduced vapor pressure coefficients at these thermal conditions necessitate extended evaporation durations, consequently prolonging phospholipid exposure to ambient oxygen, thereby initiating lipid autoxidation cascades via hydroperoxide formation [29,30]. Moreover, the elevated solution viscosity at reduced temperatures impedes thermal energy distribution through impaired convective currents [31], resulting in thermodynamic heterogeneity throughout the system. This non-uniform thermal profile leads to incomplete solvent elimination in specific regions while potentially inducing localized concentration gradients [32], ultimately compromising product homogeneity and extraction efficiency. The prolonged processing time further exacerbates phospholipid exposure to interfacial phenomena at the liquid-air boundary, potentially disrupting molecular organization through surface denaturation mechanisms [33,34].

Optimal PL-DHA/EPA recovery was achieved at 45 °C, representing the ideal equilibrium between thermodynamic efficiency and molecular stability. At this temperature, the system exhibits sufficient vapor pressure to facilitate expeditious solvent removal while remaining below the thermal threshold for significant phospholipid degradation. The moderately elevated temperature reduces solution viscosity to levels permitting optimal convective mixing, thereby ensuring uniform thermal distribution and preventing localized concentration fluctuations. The kinetic parameters governing oxidative reactions remain sufficiently suppressed at this temperature, preserving the unsaturated bonds within DHA moieties [35].

Temperature elevation beyond the optimal range (55 to 65 °C) precipitated marked diminution in PL-DHA/EPA yield through multiple deteriorative mechanisms. Elevated thermal energy accelerates oxidative degradation kinetics of polyunsaturated fatty acids, particularly docosahexaenoic acid with its six methylene-interrupted double bonds, rendering it exceptionally susceptible to free radical-mediated peroxidation. The propagation of these oxidative cascades generates lipid hydroperoxides and subsequent decomposition products, including reactive aldehydes and ketones that further compromise phospholipid integrity [36]. Concomitantly, heightened thermal conditions catalyze hydrolytic cleavage of ester linkages between glycerol and fatty acid moieties, generating lysophospholipids and free fatty acids, thereby reducing the concentration of intact phospholipid species [37].

### 3.2. Response Surface Analysis

The efficiency of PL-DHA/EPA extraction from marine sources is significantly influenced by multiple processing parameters. To elucidate the complex relationships between these variables and establish optimal extraction conditions, response surface methodology employing a Box-Behnken design was implemented. Four critical extraction parameters—liquid-to-material ratio (A), freezing temperature (B), freezing duration (C), and rotary evaporation temperature (D)—were systematically investigated at three levels each, generating 29 experimental combinations (Table 2).

**Table 2:** Response surface methodology, experimental design, and results for PL-DHA/EPA yield optimization.

Run	A	B (°C)	C (h)	D (°C)	PL-DHA/EPA Yield (g)
1	5	−20	30	45	0.2910
2	6	−20	25	45	0.3642
3	6	−20	30	55	0.2831
4	6	−20	20	55	0.2913
5	6	−30	30	45	0.2347
6	6	−30	25	55	0.2809
7	6	−20	25	45	0.3542
8	5	−20	25	35	0.3123
9	5	−30	25	45	0.2472
10	7	−30	25	45	0.2692
11	7	−20	25	35	0.3287
12	6	−30	20	45	0.2901
13	6	−20	20	35	0.3213
14	5	−20	20	45	0.2822
15	7	−10	25	45	0.1199
16	6	−20	30	35	0.3024
17	5	−10	25	45	0.1464
18	6	−20	25	45	0.3824
19	6	−10	25	55	0.1373
20	7	−20	30	45	0.2671
21	6	−30	25	35	0.221
22	6	−20	25	45	0.3756
23	6	−10	30	45	0.1483
24	6	−20	25	45	0.3923
25	6	−10	25	35	0.1535
26	7	−20	25	55	0.3324
27	6	−10	20	45	0.1529
28	5	−20	25	55	0.3129
29	7	−20	20	45	0.2769

The Box-Behnken experimental design with four independent variables—liquid-to-material ratio (A), freezing time (B), freezing temperature (C), and rotary evaporation temperature (D)—was employed to optimize PL-DHA/EPA extraction from krill oil. Response values represent the mass yield of PL-DHA/EPA (g) obtained from 10 g of krill oil starting material, calculated as the product of purified extract weight and PL-DHA/EPA concentration percentage.

Multiple regression analysis of the experimental data yielded a second-order polynomial model describing the relationship between extraction yield (Y) and the independent variables:

$$\begin{array}{cc} Y=-3.17646+ & 0.460336A+0.0954492B-0.0509484C+0.0190034D-0.00093AB-0.0012125AC\\ & +7.75\times 10^{-5}AD+0.000254BC+5.35\times 10^{-5}BD-0.00019025CD-0.03872A^{2}\\ & -0.0017733B^{2}-0.0013682C^{2}-0.000273575D^{2} \end{array}$$

The statistical significance of the model was confirmed by analysis of variance (ANOVA), revealing a coefficient of determination (R^2^) of 0.9664. The adjusted R^2^ (0.9328) and predicted R^2^ (0.8285) values demonstrated good agreement, confirming the model’s predictive capacity. The lack-of-fit test was non-significant (*p* > 0.05), further validating the model’s adequacy for navigating the design space. The yield values demonstrate substantial variation (0.1199–0.3923 g) across different parameter combinations, indicating the significant influence of processing conditions on extraction efficiency. Central point replicates (runs 2, 7, 18, 22, and 24) confirm good reproducibility with a relative standard deviation of less than 5%.

#### 3.2.1. Interaction Effects of Liquid-to-Material Ratio and Freezing Duration on PL-DHA/EPA Extraction Efficiency

**Figure 2:**
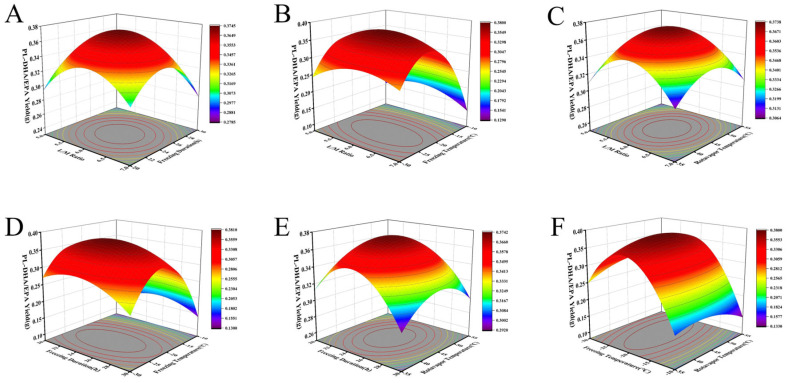
Response surface methodology analysis of interactive effects between extraction parameters on PL-DHA/EPA yield from krill oil. Three-dimensional response surface plots showing the interactive effects of (**A**) liquid-to-material ratio and freezing duration; (**B**) liquid-to-material ratio and freezing temperature; (**C**) liquid-to-material ratio and rotary evaporation temperature; (**D**) freezing duration and freezing temperature; (**E**) freezing duration and rotary evaporation temperature; and (**F**) freezing temperature and rotary evaporation temperature on PL-DHA/EPA yield. Color gradient from purple to red indicates increasing yield values, with response surfaces generated based on the second-order polynomial model. PL-DHA/EPA yield represents the weight (g) of PL-DHA/EPA obtained from 10 g of krill oil.

The response surface analysis revealed interaction between liquid-to-material ratio and freezing duration on PL-DHA/EPA extraction efficiency, manifesting through complex phase behavior and crystallization kinetics that influence separation outcomes (Figure 2A). This interaction modulates the thermodynamic and kinetic aspects of phase separation during selective crystallization. In systems with lower liquid-to-material ratios (5:1 to 6:1), elevated solute concentrations significantly alter crystallization pathways. Extended freezing durations (20 h to 25 h) become essential as increased solution viscosity impedes molecular reorganization and crystal growth [38]. Insufficient freezing periods result in incomplete phase separation characterized by numerous small crystals with substantial phospholipid entrapment within rapidly forming triglyceride crystal lattices [39]. For these concentrated systems, freezing duration exhibits a non-linear relationship with extraction efficiency, stemming from time-dependent secondary crystallization processes, particularly Ostwald ripening [40], which proceeds more slowly due to restricted molecular mobility. Consequently, concentrated extraction media require disproportionately longer freezing periods for effective phase separation.

At elevated liquid-to-material ratios (6:1 to 7:1), extended freezing durations (beyond 25 h) demonstrate diminishing returns on extraction efficiency. Enhanced molecular mobility in dilute solutions accelerates crystallization processes, while greater mean free path between molecules facilitates more rapid crystal growth and reorganization [41]. Additionally, lower solution viscosity enhances heat transfer efficiency, allowing faster thermal equilibrium achievement throughout the sample. The interaction further manifests through combined influence on the critical nucleation-to-growth ratio. At an intermediate liquid-to-material ratio (6:1) with moderate freezing duration (25 h), nucleation rate and crystal growth velocity achieve an optimal balance, promoting formation of well-defined crystals with minimal phospholipid entrapment. This facilitates discrete crystalline phases that effectively separate from the phospholipid-rich liquid phase.

#### 3.2.2. Interaction Effects of Liquid-to-Material Ratio and Freezing Temperature on PL-DHA/EPA Extraction Efficiency

Response surface analysis revealed interactive effects between liquid-to-material ratio and freezing temperature on PL-DHA/EPA extraction efficiency, modulating phase equilibria and crystallization thermodynamics that determine separation efficacy (Figure 2B). This interaction mechanistically derives from temperature-dependent solubility dynamics modified by solvent proportions. At elevated liquid-to-material ratios (6:1 to 7:1), the system demonstrates pronounced sensitivity to freezing temperature. Moderate freezing temperatures (−20 °C) combined with these higher solvent proportions establish optimal conditions for selective crystallization, wherein neutral lipids form discrete crystals while phospholipids remain solubilized. This selective partitioning occurs as increased solvent availability reduces viscosity, facilitating ordered molecular alignment during crystallization [42].

When these higher liquid-to-material ratios encounter more extreme temperatures (−20 °C to −30 °C), excessive supercooling induces rapid, non-selective crystallization with significant phospholipid entrapment within the crystal network, reducing extraction efficiency despite abundant solvent availability [43]. Conversely, at lower liquid-to-material ratios (5:1 to 6:1), freezing temperatures between −10 °C and −20 °C yield poor separation due to insufficient thermodynamic driving force for phase differentiation. Elevated solute concentration increases viscosity, impeding molecular diffusion necessary for organized crystallization. However, at lower temperatures (−25 °C to −30 °C), enhanced thermodynamic driving force partially compensates for restricted molecular mobility, improving separation selectivity [44]. The interaction additionally influences colligative properties, particularly freezing point depression. Higher liquid-to-material ratios dilute solute concentration, reducing these effects and creating non-linear response patterns across the experimental domain.

#### 3.2.3. Interaction Effects of Liquid-to-Material Ratio and Rotary Evaporation Temperature on PL-DHA/EPA Extraction Efficiency

Response surface analysis revealed interactive effects between liquid-to-material ratio (5:1 to 7:1) and rotary evaporation temperature (35 °C to 55 °C) on PL-DHA/EPA extraction efficiency (Figure 2C). This interaction profoundly influences solvent-solute dynamics, molecular conformational stability, and separation selectivity through interdependent physicochemical mechanisms. At lower liquid-to-material ratios (5:1), extraction efficiency exhibits sensitivity to rotary evaporation temperature. When combined with moderate temperatures (35 °C to 45 °C), these concentrated systems demonstrate limited extraction efficiency due to insufficient phospholipid solubilization. The restricted solvent volume creates localized saturation zones that impede the complete dissolution of phospholipid components [45]. However, when these concentrated conditions are coupled with elevated evaporation temperatures (45 °C to 55 °C), enhanced molecular mobility and reduced solvent viscosity partially compensate for limited solvent availability, facilitating improved phospholipid diffusion from the lipid matrix into the solvent phase [11].

Conversely, at higher liquid-to-material ratios (6:1 to 7:1), the interaction manifests through different mechanistic pathways. These dilute systems exhibit optimal extraction efficiency when paired with moderate evaporation temperatures (40 °C to 45 °C), creating ideal conditions for selective phospholipid solubilization while minimizing thermal degradation. The abundant solvent availability ensures sufficient concentration gradient for effective mass transfer, while moderate temperatures maintain structural integrity of the polyunsaturated fatty acid moieties within phospholipids. However, when these higher solvent ratios are combined with elevated temperatures (50 °C to 55 °C), a counterproductive phenomenon emerges wherein accelerated oxidative degradation and hydrolytic reactions compromise phospholipid stability despite efficient initial extraction [46,47].

#### 3.2.4. Interaction Effects of Freezing Duration and Freezing Temperature on PL-DHA/EPA Extraction Efficiency

Response surface analysis revealed a profound interaction between freezing duration (20–30 h) and temperature (−10 °C to −30 °C) as the predominant factor influencing PL-DHA/EPA extraction efficiency (Figure 2D). This interaction governs differential crystallization phenomena based on disparate freezing points of triglycerides and phospholipids in ethanolic solution, determining separation efficacy and extract purity.

The mechanistic foundation lies in the differential solubility behavior of lipid classes in anhydrous ethanol across thermal conditions and exposure durations. Triglycerides exhibit higher freezing points in ethanolic solutions compared to phospholipids due to their neutral character and limited polar interactions with the solvent [48]. At −20 °C, triglycerides undergo selective crystallization while phospholipids remain solubilized, creating the fundamental basis for separation, with temporal dimensions proving critical for complete phase differentiation.

At moderate freezing temperatures (−20 °C) maintained for moderate durations (25 h), optimal separation occurs through complete triglyceride crystallization while phospholipids remain predominantly in solution. These conditions provide sufficient time for triglyceride molecules to overcome kinetic energy barriers to nucleation and crystal growth, while the temperature remains insufficient to induce phospholipid precipitation [49]. When moderate temperatures are combined with insufficient durations (<25 h), incomplete triglyceride crystallization reduces separation efficiency [50]. The nucleation and growth of triglyceride crystals follow time-dependent kinetics requiring sufficient duration for completion, with premature termination resulting in partial triglyceride retention in the liquid phase [51].

At lower freezing temperatures (−25 °C to −30 °C), diminished selectivity occurs as these conditions approach the freezing point threshold of phospholipids in ethanolic solution, resulting in partial co-precipitation. Extended periods at these temperatures induce significant phospholipid loss through undesired crystallization. Conversely, at higher temperatures (−10 °C to −15 °C), triglyceride crystallization proceeds inefficiently, requiring substantially extended durations yet still resulting in incomplete separation. The interaction further influences separation efficiency through its impact on crystal morphology and filtration characteristics.

#### 3.2.5. Interaction Effects of Freezing Duration and Rotary Evaporation Temperature on PL-DHA/EPA Extraction Efficiency

Response surface analysis revealed an interaction between freezing duration (20–30 h) and rotary evaporation temperature (35–55 °C) as a factor of PL-DHA/EPA extraction efficiency (Figure 2E). This interaction bridges crystallization-based separation with solvent removal, influencing both separation selectivity and thermal preservation of phospholipids.

The extraction process initiates with cold crystallization, wherein triglycerides selectively precipitate while phospholipids remain solubilized. Freezing duration exhibits a non-linear relationship with separation efficacy, peaking precisely at 25 h—a critical equilibrium point in the crystallization process. At shorter durations (20–25 h), incomplete triglyceride crystallization results in residual neutral lipid contamination. Conversely, extended periods (25–30 h) induce partial phospholipid precipitation and co-crystallization, reducing phospholipid recovery despite apparent thorough separation.

The optimal 25 h duration establishes an ideal phase equilibrium where triglyceride crystallization reaches completion without initiating phospholipid precipitation, allowing sufficient time for triglyceride nucleation and crystal growth while preventing phospholipids from approaching their solubility limits in the increasingly purified ethanolic solution. Following filtration, the phospholipid-enriched filtrate undergoes rotary evaporation, with temperature directly influencing molecular integrity. When 25 h freezing precedes moderate evaporation (45 °C), the system achieves maximal extraction efficiency. This specific duration provides an ideally enriched solution for evaporation, while moderate temperatures preserve molecular integrity by minimizing thermal degradation of polyunsaturated fatty acid moieties.

#### 3.2.6. Interaction Effects of Freezing Temperature and Rotary Evaporation Temperature on PL-DHA/EPA Extraction Efficiency

Response surface analysis revealed an interaction between freezing temperature (−30 °C to −10 °C) and rotary evaporation temperature (35 °C to 55 °C) as a factor of PL-DHA/EPA extraction efficiency (Figure 2F). This interaction bridges the selective crystallization phase with subsequent solvent removal, synergistically influencing both separation selectivity and molecular integrity preservation.

The mechanistic foundation of this interaction lies in the temperature-dependent phase equilibria during crystallization and subsequent thermal stability during concentration. At optimal freezing temperature (−20 °C), triglycerides selectively crystallize while phospholipids remain predominantly solubilized due to differential freezing points in ethanolic solution. When this optimally separated mixture subsequently undergoes evaporation at moderate temperature (45 °C), the system preserves molecular integrity while efficiently removing solvent, maximizing both yield and quality.

The interaction manifests distinctively across the experimental domain. At lower freezing temperatures (−30 °C to −25 °C), excessive supercooling induces non-selective co-precipitation of phospholipids alongside triglycerides due to reduced molecular mobility and diminished solvent-solute interactions [52]. When these sub-optimally separated mixtures undergo moderate evaporation (45 °C), the reduced phospholipid content in the filtrate cannot be remediated, resulting in diminished yield despite careful thermal treatment. Conversely, when these excessively cold-treated mixtures undergo higher-temperature evaporation (50–55 °C), the combination of initial phospholipid loss with subsequent thermal degradation produces significantly compromised extracts with both reduced yield and quality.

At higher freezing temperatures (−15 °C to −10 °C), insufficient crystallization driving force results in incomplete triglyceride precipitation, yielding filtrates with substantial neutral lipid contamination [53]. When these impure filtrates undergo low-temperature evaporation (35–40 °C), the result is a product with compromised purity despite minimal thermal degradation. However, when combined with elevated evaporation temperatures (50–55 °C), a compensatory effect emerges wherein thermal-induced viscosity reduction facilitates partial secondary separation during the early stages of evaporation [54], partially mitigating the initial poor separation, though still yielding suboptimal results.

### 3.3. PL-DHA/EPA Content and Phospholipid Composition Analysis

**Figure 3:**
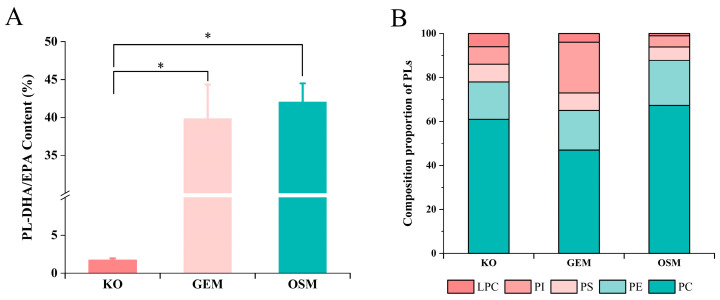
Comparative analysis of different extraction methodologies. (**A**) PL-DHA/EPA content among krill oil (KO), green extraction method (GEM), and organic solvent method (OSM). (**B**) Compositional proportion of phospholipid classes showing relative abundance of phosphatidylcholine (PC), phosphatidylethanolamine (PE), phosphatidylserine (PS), phosphatidylinositol (PI), and Lysophosphatidylcholine (LPC) across extraction methods. Error bars represent standard deviation (*n* = 3). Symbol (*) indicates significant differences (*p* < 0.05) among extraction methods determined by one-way ANOVA followed by Duncan’s multiple range test.

Comprehensive characterization of PL-DHA/EPA content and phospholipid class distribution revealed striking differences between krill oil and the fractions obtained through alternative extraction methodologies (Figure 3). Quantitative analysis demonstrated that the PL-DHA/EPA content in krill oil was merely 1.72%, reflecting the complex lipid composition of the unprocessed marine material, where neutral lipids predominate. In contrast, both extraction methods yielded substantial enrichment, with the conventional organic solvent extraction method and the green extraction method achieving impressive PL-DHA/EPA concentrations of 42.39% and 39.40% (Figure 3A).

Statistical analysis revealed no significant difference between the two extraction methodologies (*p* > 0.05), indicating that the green approach achieves comparable phospholipid enrichment efficiency despite fundamentally different physicochemical principles. The conventional organic solvent system demonstrated a slight numerical advantage, potentially attributable to the superior solvation capacity of the chloroform-methanol mixture. This binary solvent system creates a wide polarity spectrum capable of disrupting various lipid-lipid and lipid-protein interactions within the complex marine matrix [55]. However, the green methodology’s performance approaches this benchmark through distinct molecular mechanisms, leveraging ethanol’s selective solubilization properties and the differential crystallization behavior of lipid classes during controlled freezing.

Analysis of phospholipid class distribution (Figure 3B) further elucidated the extraction dynamics. Both methodologies substantially altered the phospholipid profile compared to krill oil, but without significant differences between the extraction approaches. The conventional organic solvent extraction method’s marginally higher phosphatidylcholine extraction may contribute to its slight numerical advantage in total PL-DHA/EPA content, as PC typically represents the predominant phospholipid fraction in krill oil. The comparable performance of the green methodology, despite using significantly less hazardous solvents, likely stems from the precise optimization of extraction parameters through response surface methodology, which established ideal conditions for selective phospholipid isolation.

### 3.4. Principal Component Analysis

**Figure 4:**
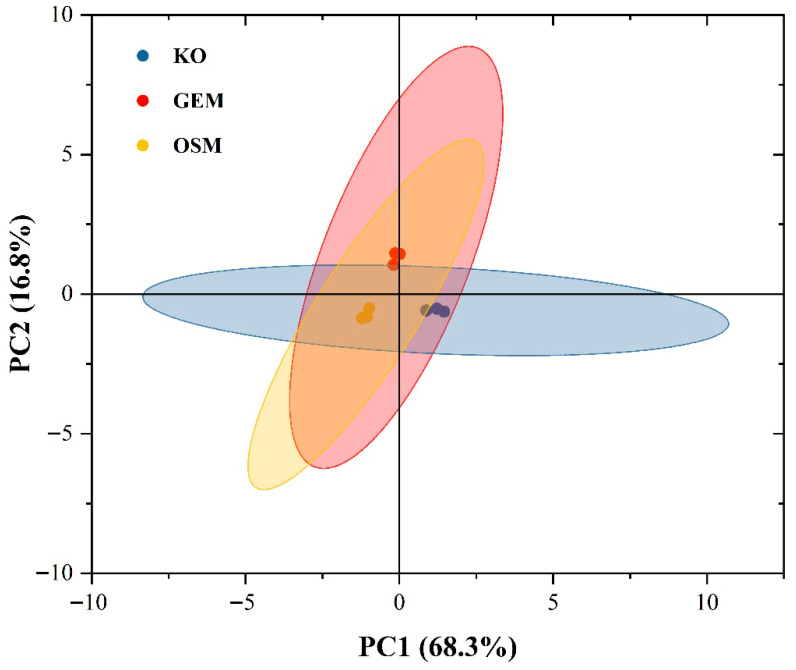
Principal Component Analysis (PCA) of phospholipid profiles from different extraction methodologies. Score plot displaying the distribution of samples along the first two principal components (PC1 and PC2), which collectively account for 85.1% of total variance. Samples are categorized into three groups: krill oil (blue circles), green extraction method (red circles), and conventional organic solvent extraction method (yellow circles). The 95% confidence ellipses demonstrate substantial overlap among all three sample groups, indicating remarkable conservation of phospholipid compositional patterns despite different extraction approaches. PC1 (68.3% variance) primarily reflects phosphatidylcholine and phosphatidylethanolamine species containing polyunsaturated fatty acids, while PC2 (16.8% variance) is influenced by lysophospholipid content.

Principal Component Analysis (PCA) was conducted to evaluate holistic variations in phospholipid profiles among krill oil and phospholipid fractions obtained through either green extraction method or conventional organic solvent extraction. The resulting score plot (Figure 4) revealed substantial overlap in the 95% confidence intervals among all three sample groups, indicating remarkable similarity in their overall phospholipid composition patterns despite different extraction methodologies. This unexpected outcome contrasts with the distinct variations observed in extraction yields and individual phospholipid species distributions previously discussed.

The first two principal components collectively explained 85.1% of the total variance (PC1: 68.3%; PC2: 16.8%), suggesting robust data reduction. The substantial overlap in confidence ellipses indicates that both extraction methodologies effectively preserve the core phospholipid signature of the parent krill oil, maintaining critical structural characteristics and molecular species distribution.

This phospholipid profile conservation is particularly noteworthy given the distinct physicochemical principles underlying the two extraction approaches. The green extraction method, leveraging selective ethanol solubilization and low-temperature precipitation, appears to maintain similar extraction selectivity as the conventional chloroform-methanol system despite fundamentally different solvent polarities and interaction mechanisms. The molecular basis for this conservation likely involves the dominance of phospholipid headgroup characteristics in determining extraction behavior over the influence of acyl chain variations. These findings suggest that the green extraction method achieves compositional equivalence to conventional organic extraction while offering enhanced sustainability and reduced toxicity concerns.

### 3.5. Molecular Species Distribution Analysis

**Figure 5:**
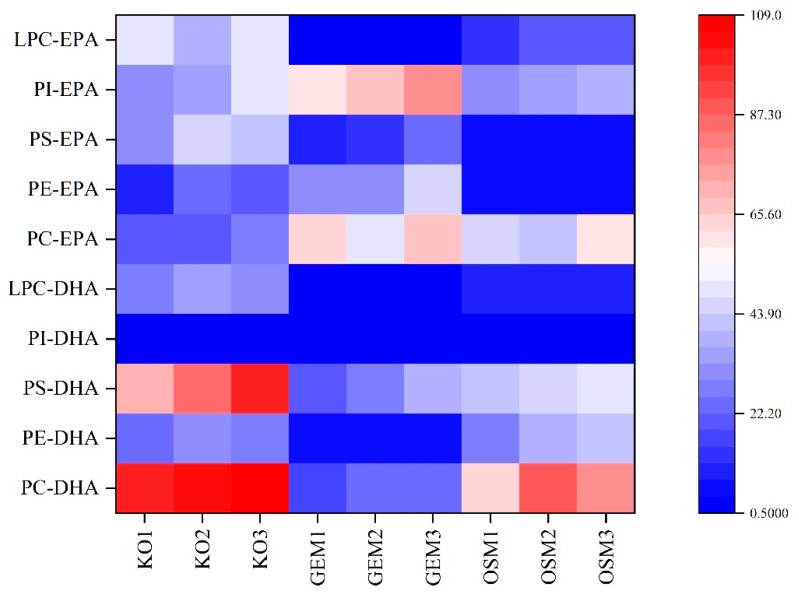
Heatmap analysis of phospholipid class-specific DHA/EPA distribution across extraction methodologies. The heatmap displays the relative abundance of DHA and EPA in five major phospholipid classes: phosphatidylcholine (PC-DHA/EPA), phosphatidylethanolamine (PE-DHA/EPA), phosphatidylserine (PS-DHA/EPA), phosphatidylinositol (PI-DHA/EPA), and lysophosphatidylcholine (LPC-DHA/EPA) extracted through krill oil (KO), green extraction method (GEM), and organic solvent method (OSM).

Hierarchical clustering analysis of phospholipid molecular species distribution revealed distinctive extraction-dependent patterns in fatty acid incorporation (Figure 5). The heatmap visualization illustrates remarkable differences in phospholipid profiles among krill oil, conventional organic solvent extraction, and green extraction method products, providing insights into the selective mechanisms governing different extraction approaches. Color intensity represents normalized content levels, with red indicating high abundance and blue indicating low abundance.

Notably, krill oil and organic solvent extraction method products exhibited similar phospholipid species distributions, with both demonstrating enrichment in PL-DHA molecular species, particularly PC-DHA and PS-DHA. This similarity suggests that conventional organic extraction maintains the native phospholipid profile of the raw material with minimal selective discrimination between molecular species. The chloroform-methanol system’s broad polarity spectrum enables comprehensive solubilization of the entire phospholipid fraction, functioning essentially as a scaled representation of the original phospholipid distribution. This non-selective pattern likely stems from the complementary polarities of the binary solvent system, which accommodates diverse phospholipid structures regardless of fatty acid composition or headgroup characteristics [56].

In contrast, the green extraction method demonstrated a distinctly different molecular signature, with significant enrichment in EPA-containing phospholipids, particularly PC-EPA and PI-EPA species. This selective extraction profile reveals sophisticated molecular discrimination mechanisms operating during ethanol-based extraction. The preferential extraction of EPA-containing phospholipids over their DHA counterparts can be attributed to subtle differences in molecular geometry and polarity. EPA’s five double bonds (versus DHA’s six) create slightly higher polarity and increased molecular linearity, enhancing solvation efficiency in ethanol’s intermediate polarity environment [57]. This effect is particularly pronounced for phosphatidylinositol species, where the multiple hydroxyl groups on the inositol ring form favorable hydrogen bonds with ethanol, further enhancing extraction selectivity.

This temperature-dependent separation mechanism demonstrates particular effectiveness for PI-EPA, where the bulky inositol headgroup creates steric hindrance that inhibits ordered packing in crystalline structures. This unintended but potentially beneficial molecular selectivity represents a unique advantage of the green extraction method, highlighting that alternative extraction approaches may yield products with distinctive molecular signatures offering novel functional properties beyond simple phospholipid enrichment.

### 3.6. Oxidative Stability Assessment

**Figure 6:**
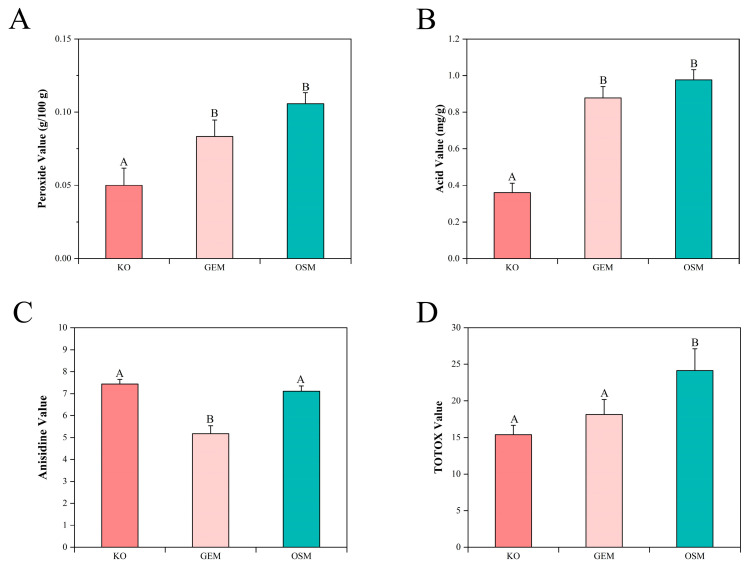
Oxidative stability assessment of extracted PL-DHA/EPA across different methodologies. (**A**) Peroxide values indicating primary oxidation levels in krill oil (KO), green extraction method (GEM), and organic solvent method (OSM) samples, expressed as grams of iodine per 100 g of lipid (g/100 g). (**B**) Acid values representing free fatty acid content resulting from hydrolytic rancidity, expressed as milligrams of potassium hydroxide per gram of lipid (mg/g). (**C**) Anisidine values quantifying secondary oxidation products, particularly aldehydic compounds, expressed as anisidine units. (**D**) Total oxidation (TOTOX) values calculated as 2 × PV + AV, providing a comprehensive oxidative deterioration assessment combining primary and secondary oxidation parameters. Error bars represent standard deviation (*n* = 3). Different letters above bars indicate significant differences (*p* < 0.05) among extraction methods determined by one-way ANOVA followed by Duncan’s multiple range test.

Comprehensive evaluation of oxidative stability parameters revealed distinctive patterns across krill oil and phospholipid fractions obtained through alternative extraction methodologies (Figure 6). These differential oxidation profiles provide mechanistic insights into extraction-dependent effects on both primary and secondary oxidation pathways.

#### 3.6.1. Peroxide Values Analysis

Primary oxidation, as evidenced by peroxide values, demonstrated the lowest susceptibility in krill oil samples, followed by the green extraction method and organic solvent extraction (Figure 6A). The superior oxidative resistance of krill oil can be attributed to the synergistic natural antioxidant matrix [58], particularly astaxanthin and endogenous tocopherols, which effectively scavenge hydroxyl radicals through sequential electron transfer mechanisms and interrupt propagation chain reactions via hydrogen atom donation to lipid peroxyl radicals [59]. Astaxanthin’s conjugated polyene structure provides exceptional radical quenching capacity through resonance stabilization of the resulting radical cation [60], while α-tocopherol regeneration occurs through ascorbic acid-mediated reduction cycles that maintain continuous antioxidant protection [61].

The phospholipid structure itself contributes fundamentally to oxidative stability through molecular organization principles, where the zwitterionic polar head groups of phosphatidylcholine and phosphatidylethanolamine create hydrated microenvironments that sterically hinder molecular oxygen diffusion to the polyunsaturated fatty acid chains at the sn-2 position [62]. Green extraction method preserve native phospholipid bilayer architecture through precisely controlled temperature conditions that prevent thermal-induced conformational changes in phospholipid headgroup orientation. The mild processing environment maintains the integrity of phospholipid-protein complexes and associated antioxidant binding sites [63], preserving the critical packing density that shields vulnerable bis-allylic hydrogen positions from oxidative attack. Consequently, the lamellar organization characteristic of native marine phospholipids remains intact, providing enhanced protection against lipid peroxidation initiation compared to disrupted membrane structures resulting from organic solvent extraction conditions.

#### 3.6.2. Acid Values Analysis

Acid values mirrored the peroxide value trends, with krill oil maintaining the lowest acid values, while green extraction and organic solvent extraction methods showed elevated but statistically comparable levels (Figure 6B). This parallel relationship indicates that hydrolytic rancidity proceeds concurrently with oxidative processes, likely facilitated by trace metal contamination and residual moisture in the extracted samples. The enzymatic and thermal stress inherent in extraction processes promotes phospholipase A_2_ activation, catalyzing the hydrolysis of phospholipid ester bonds and generating free fatty acids [64,65]. Organic solvent extraction method induces significant disruption of phospholipid membrane integrity through dehydration and lipid-protein complex dissociation, exposing previously protected ester linkages to hydrolytic enzymes [66]. Conversely, green extraction maintains hydrated phospholipid vesicles that resist enzymatic penetration, thereby limiting hydrolytic degradation despite comparable processing times.

#### 3.6.3. P-Anisidine Values Analysis

Secondary oxidation products, quantified through anisidine values, revealed an unexpected reversal in the oxidation pattern, with green extraction method yielding the lowest aldehydic compound concentrations, followed by organic solvent extraction, and krill oil showing the highest values (Figure 6C). This paradoxical finding reflects the advanced oxidative state of krill oil during commercial processing and storage, where primary oxidation products have undergone extensive decomposition to form secondary aldehydes, particularly 2,4-dienals and saturated aldehydes [67]. The structural modifications induced by different extraction methodologies significantly influence aldehyde formation kinetics. Organic solvent extraction disrupts phospholipid headgroup orientation and intermolecular hydrogen bonding, facilitating the migration of lipid hydroperoxides and their subsequent decomposition through β-scission reactions [68]. Green extraction preserves the lamellar phospholipid organization and maintains critical water content within the polar regions, creating a compartmentalized system that restricts radical diffusion and limits aldehyde propagation. The Schiff base formation between amino groups in phosphatidylserine and phosphatidylethanolamine with aldehydic carbonyls further modulates the apparent anisidine values [69], with intact phospholipid structures promoting more extensive cross-linking reactions that sequester reactive aldehydes from detection.

#### 3.6.4. TOTOX Values Analysis

The Total Oxidation (TOTOX) values, calculated as 2 × PV + AV, provided a comprehensive assessment of overall oxidative deterioration, revealing complex interactions between primary and secondary oxidation pathways (Figure 6D). Krill oil samples exhibited the lowest TOTOX values, demonstrating superior overall oxidative stability despite elevated secondary oxidation products. This apparent paradox reflects the dominant contribution of exceptionally low peroxide values, which are weighted doubly in TOTOX calculations, effectively compensating for moderate anisidine levels. The natural antioxidant matrix, particularly astaxanthin and endogenous tocopherols, maintained robust protection against primary oxidation initiation, while secondary aldehydic compounds represented historical oxidative events during commercial processing rather than active deterioration.

The green extraction method demonstrated intermediate TOTOX values, reflecting the balanced oxidative profile achieved through structural preservation mechanisms. Despite moderate primary oxidation levels, the exceptionally low anisidine values contributed to favorable overall oxidation indices. This optimization resulted from preservation of enzymatic antioxidant systems, particularly glutathione peroxidase and catalase activities, which catalyze hydroperoxide reduction to corresponding alcohols without aldehyde formation.

Organic solvent extraction method yielded the highest TOTOX values, reflecting cumulative deterioration from both elevated primary and secondary oxidation pathways. Aggressive extraction conditions disrupted natural antioxidant networks through selective partitioning of hydrophilic protective compounds, leaving lipophilic components vulnerable to comprehensive oxidative attack. Chloroform-methanol systems induced conformational changes exposing polyunsaturated fatty acid chains while eliminating protective water shells, facilitating both hydroperoxide formation and subsequent aldehydic decomposition [70].

## 4. Conclusions

This investigation successfully developed a novel environmentally-friendly methodology for the selective extraction and enrichment of PL-DHA/EPA from krill oil through differential temperature-controlled crystallization in ethanolic systems. The fundamental thermodynamic principle exploiting crystallization behavior differences between phospholipids and triacylglycerols demonstrated remarkable selectivity, achieving high-purity PL-DHA/EPA recovery while maintaining structural integrity of bioactive phospholipid complexes.

The sustainable ethanol-based extraction system eliminated environmental concerns associated with chlorinated solvents while achieving enhanced product quality through temperature-programmed crystallization protocols. Response surface methodology optimization established critical processing parameters that maximize PL-DHA/EPA recovery. The methodology’s scalability and economic viability position it as a viable alternative for industrial marine phospholipid purification, addressing critical market demands for high-quality omega-3 products with enhanced bioavailability characteristics. Comprehensive oxidative stability analysis revealed superior performance of the developed methodology compared to conventional organic solvent extraction, with significantly reduced TOTOX values and preserved antioxidant matrix integrity.

## Data Availability

The original contributions presented in this study are included in the article. Further inquiries can be directed to the corresponding author. The data presented in this study are available on request from the corresponding author due to (specify the reason for the restriction).
